# Integration of MicroRNAs with nanomedicine: tumor targeting and therapeutic approaches

**DOI:** 10.3389/fcell.2025.1569101

**Published:** 2025-04-07

**Authors:** Pelin Telkoparan-Akillilar, Silvia Chichiarelli, Paolo Tucci, Luciano Saso

**Affiliations:** ^1^ Department of Medical Biology, Faculty of Medicine, Gazi University, Ankara, Türkiye; ^2^ Department of Biochemical Sciences “A. Rossi-Fanelli”, Sapienza University of Rome, Rome, Italy; ^3^ Department of Clinical and Experimental Medicine, University of Foggia, Foggia, Italy; ^4^ Department of Physiology and Pharmacology “Vittorio Erspamer”, La Sapienza University, Rome, Italy

**Keywords:** MicroRNA (miRNA), nanomedicine, tumor targeting, nanoparticle delivery systems, cancer therapy

## Abstract

MicroRNAs (miRNAs) are small, non-coding RNA molecules that play a pivotal role in the post-transcriptional regulation of gene expression. Over the past decade, they have emerged as key regulators in cancer progression, influencing different cellular processes such as proliferation, apoptosis, metastasis, and immune evasion. Their unique ability to target multiple genes simultaneously makes miRNAs highly attractive as potential therapeutic agents in oncology. However, several challenges have hindered their direct clinical application, most notably their inherent instability in biological fluids, rapid degradation by nucleases, and inefficient delivery to specific tumor sites. Additionally, off-target effects and the potential for toxicity further complicate the therapeutic use of miRNAs. Nanomedicine offers a promising solution to these challenges by enabling the development of advanced platforms for the stable, safe, and targeted delivery of miRNAs. Nanoparticle-based delivery systems, such as liposomes, polymeric nanoparticles, and inorganic nanocarriers, can protect miRNAs from degradation, improve their bioavailability, and allow for precise tumor targeting through passive or active targeting mechanisms. These nanocarriers can also be engineered to release miRNAs in response to specific stimuli within the tumor microenvironment, enhancing therapeutic efficacy while minimizing side effects. This review will explore the integration of miRNAs with nanotechnology, focusing on various nanoparticle formulations and their roles in enhancing miRNA stability, specificity, and function in cancer treatment. In addition, we will discuss current advances in preclinical and clinical applications, highlight promising tumor-targeting strategies, and address the remaining challenges such as toxicity, immunogenicity, and scalability. Future research should focus on overcoming these barriers, ultimately paving the way for the widespread adoption of personalized miRNA-based nanomedicine in cancer therapy.

## 1 Introduction

Nanomedicine, the application of nanotechnology in healthcare, is an emerging and transformative field that holds immense potential to revolutionize the treatment of various diseases, particularly cancer. Nanomedicine utilizes nanoscale materials (ranging from 1 to 100 nm) with unique physical, chemical, and biological properties, enabling highly targeted and efficient therapeutic approaches (Shi et al., 2017). The central idea of nanomedicine is to harness the versatility of nanoparticles (NPs) to deliver drugs, nucleic acids, proteins, and other therapeutic agents directly to the site of disease, minimizing systemic toxicity and improving treatment efficacy (Peer et al., 2007; Farokhzad and Langer, 2009). While conventional cancer therapies such as chemotherapy, radiotherapy, and surgery have significantly advanced cancer treatment, they often come with numerous limitations, including non-specific drug distribution, systemic toxicity, off-target effects, and the development of drug resistance (Fan et al., 2023). Traditionally, nanomedicines have been used to regulate the biodistribution and target site accumulation of systemically administered chemotherapeutic drugs, thereby improving the balance between efficacy and toxicity. In preclinical studies, nanomedicines have been shown to inhibit tumor growth and prolong survival; however, in clinical applications, patients often benefit from them mainly due to reduced or altered side effects rather than significantly improved efficacy (van der Meel et al., 2019). Nanomedicine addresses these challenges by improving the precision of drug delivery, increasing bioavailability, and minimizing side effects, ultimately leading to better patient outcomes.

Nanoparticles serve as the primary vehicles for drug delivery in nanomedicine, and their inherent properties, such as a high surface area-to-volume ratio, biocompatibility, and the ability to carry diverse therapeutic agents, make them ideal candidates for cancer therapy (Dang and Guan, 2020). The versatility of nanoparticles allows them to encapsulate a wide range of therapeutic agents, including traditional chemotherapy drugs, small molecules, nucleic acids, and biologics. These agents can be delivered directly to the tumor site, thus increasing drug bioavailability at the targeted location while reducing unwanted exposure to healthy tissues. Moreover, nanoparticles can be engineered with surface modifications that allow for targeted delivery, taking advantage of molecular interactions such as ligand-receptor binding or the enhanced permeability and retention (EPR) effect, a characteristic phenomenon in solid tumors that allows NPs to accumulate more readily at the tumor site (Yao et al., 2020).

One of the most promising therapeutic agents that has gained significant attention in the context of cancer therapy is microRNAs (miRNAs). miRNAs are small, non-coding RNA molecules that regulate gene expression post-transcriptionally by binding to complementary sequences in messenger RNAs (mRNAs), leading to mRNA degradation or inhibition of translation (Bartel, 2009). These regulatory molecules are involved in a broad range of cellular processes, such as cell proliferation, differentiation, apoptosis, and metabolism, making them pivotal in maintaining cellular homeostasis (van Wijk et al., 2022). In the context of cancer, miRNAs have been found to play crucial roles in both tumor suppression and oncogenesis, depending on their expression patterns. Dysregulation of miRNA expression is a hallmark of many cancers, with some miRNAs functioning as oncogenes (oncomiRs), while others act as tumor suppressors (Esquela-Kerscher and Slack, 2006; Kasinski and Slack, 2011; Di Leva et al., 2014). As a result, miRNAs represent promising therapeutic targets for cancer treatment, offering the potential to modulate key pathways that drive tumorigenesis.

Despite the promise of miRNA-based therapies, the clinical translation of these strategies has been limited by several factors, the most significant of which is the efficient delivery of miRNAs to the tumor site. Due to their small size, miRNAs are highly susceptible to degradation by ribonucleases in the bloodstream, reducing their stability and bioavailability (Balzano et al., 2015; Glinge et al., 2017). Furthermore, the ability of free miRNAs to effectively cross biological barriers and reach the intended target cells in tumors is restricted. To address these challenges, nanotechnology has emerged as an ideal solution. Nanoparticles can protect miRNAs from enzymatic degradation, increase their stability in circulation, and enhance their delivery efficiency by facilitating their entry into target cells. Moreover, the surface of nanoparticles can be functionalized with targeting ligands, allowing for the specific delivery of miRNAs to cancer cells, thus minimizing off-target effects and increasing therapeutic efficacy (Lee et al., 2019).

The combination of nanotechnology and miRNA-based therapies offers a powerful synergistic approach to overcome the inherent limitations of conventional cancer treatments. By using nanoparticles to deliver miRNAs, the bioavailability and stability of miRNAs are significantly improved, and their therapeutic potential can be fully realized. This approach also offers the possibility of personalized cancer therapy, as the expression profiles of miRNAs in individual tumors can be used to tailor treatments to the specific molecular and genetic characteristics of cancer (Pagoni et al., 2023). The development of nanoparticle-based miRNA delivery systems holds tremendous promise in the quest for more precise, effective, and less toxic cancer treatments.

## 2 Mechanisms of miRNA in cancer

**FIGURE 1:**
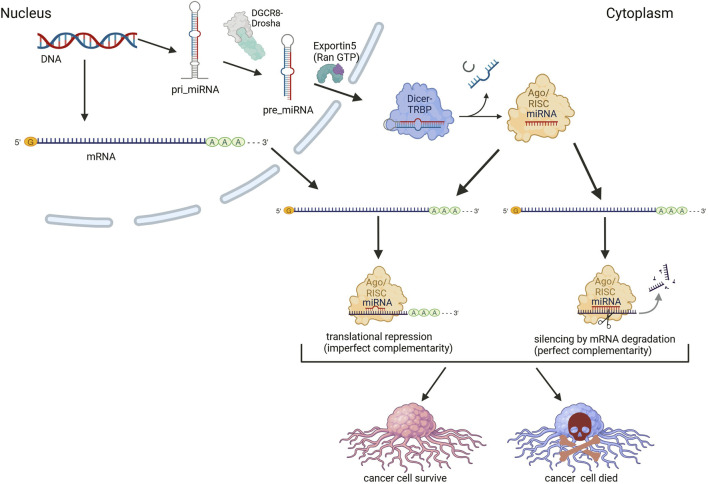
Mechanism of miRNA-mediated regulation of target mRNA translation in cancer cells. Messenger RNA (mRNA) and miRNA are transcribed by RNA polymerases (II, and/or III only for miRNA). The primary miRNA (pri-miRNA) is processed by the Drosha-DGCR8 complex. The pre-miRNA is then actively exported from the nucleus to the cytoplasm through the Exportin-5–Ran/GTP transport system. In the cytoplasm, the pre-miRNA undergoes processing by the Dicer enzyme in association with the TAR RNA-binding protein (TRBP), generating a mature miRNA duplex. The guide strand is loaded onto the Argonaute (Ago) protein, forming the RNA-induced silencing complex (RISC). The other strand of the duplex is typically degraded. The functional miRNA-RISC complex binds to complementary sequences in the 3′ untranslated region (3′ UTR) of target mRNAs, leading to either translational repression or mRNA degradation. The specific outcome depends on the degree of sequence complementarity: Imperfect complementarity results in translational silencing, preventing the production of the corresponding protein; Perfect or near-perfect complementarity leads to mRNA cleavage and subsequent degradation, reducing gene expression at the transcript level. miRNAs can act as oncogenic miRNAs (oncomiRs) or tumor-suppressor miRNAs, depending on their target genes. OncomiRs suppress tumor suppressor genes, promoting cancer cell proliferation and survival, while tumor-suppressor miRNAs inhibit oncogenes, thereby preventing tumor growth. This dual nature of miRNA function highlights their critical role in cancer biology, where dysregulation of miRNA expression can dictate cell fate. In this respect miRNA-mediated regulation represents a potential avenue for therapeutic intervention, with strategies aimed at restoring physiological miRNA function. Created in BioRender. Chichiarelli, S. (2025) https://BioRender.com/b38z967.

miRNAs are small, single-stranded RNA molecules approximately 20–22 nucleotides in length that play a crucial role in the post-transcriptional regulation of gene expression. They regulate gene expression by binding to complementary sequences within the 3′ untranslated regions (UTRs) of target mRNAs, leading to either mRNA degradation or inhibition of translation (Bartel, 2009). miRNAs control a wide range of biological processes, including development, apoptosis, differentiation, and cell cycle regulation, all of which are essential for maintaining cellular homeostasis and proper tissue function. Given their involvement in these fundamental cellular processes, it is not surprising that miRNAs are also deeply implicated in cancer biology (Figure 1).

### 2.1 miRNAs as oncogenes and tumor suppressors in cancer

In the context of cancer, miRNAs can function as oncogenes or tumor suppressors, depending on their target genes and the cellular context. When miRNAs act as oncogenes, they are commonly referred to as oncomiRs, as they contribute to tumorigenesis by silencing tumor-suppressive genes or promoting cell proliferation (Esquela-Kerscher and Slack, 2006). On the other hand, tumor-suppressive miRNAs act to inhibit cancer progression by targeting oncogenes and other molecules involved in tumor growth (Otmani and Lewalle, 2021). Dysregulation of miRNA expression, whether through overexpression of oncomiRs or downregulation of tumor-suppressive miRNAs, can lead to uncontrolled cell proliferation, evasion of apoptosis, and metastasis, hallmarks of cancer (Rajasegaran et al., 2021). Given their pivotal role in tumorigenesis, miRNAs have emerged as promising therapeutic targets, with strategies focusing on restoring the function of tumor-suppressive miRNAs through miRNA mimics or inhibiting oncogenic miRNAs using antimiRs. miRNA mimics and molecules targeting miRNAs (antimiRs) have shown promise in the preclinical development stage, and several miRNA-targeted therapeutic approaches have reached clinical development, including a tumor-suppressing miRNA mimic that has entered phase I clinical trials for cancer treatment and an antimiR targeting a specific miRNA that has reached phase II trials for hepatitis treatment. These advancements highlight the therapeutic potential of miRNA-based interventions in modulating cancer progression and other diseases (Rupaimoole and Slack, 2017).

One of the most well-known oncomiRs is miR-21, which is frequently upregulated in a variety of cancers, including breast cancer, lung cancer, and colorectal cancer (Iorio et al., 2005; Markou et al., 2008; Schetter et al., 2008). miR-21 promotes tumorigenesis by suppressing key tumor suppressors genes, such as PTEN (phosphatase and tensin homolog), PDCD4 (programmed cell death 4), and TIMP3 (tissue inhibitor of metalloproteinases 3) (Gaur et al., 2011; Del Campo et al., 2015; Wu et al., 2017). These genes play critical roles in regulating cell cycle progression, apoptosis, and metastasis, and their inhibition by miR-21 contributes to enhanced cell proliferation, migration, and invasion, as well as resistance to chemotherapy. miR-21’s involvement in various cancer types, coupled with its ability to promote tumorigenesis, underscores its potential as both a diagnostic biomarker and a therapeutic target for cancer treatment.

Similarly, miR-155 has been identified as another oncomiR that is overexpressed in a range of cancers, including lymphoma, breast cancer, and gastric cancer (Gao et al., 2017; Qu et al., 2018; Zheng et al., 2020). miR-155 promotes tumorigenesis by targeting key regulators of immune responses and apoptosis, such as SOCS1 (suppressor of cytokine signaling 1) and TP53 (tumor protein p53) (Gironella et al., 2007; Zhang et al., 2011). Its overexpression is associated with increased proliferation and metastasis, and it has been shown to contribute to immune evasion by modulating immune cell functions, making it an attractive target for therapeutic interventions (Vigorito et al., 2013; Qu et al., 2015).

On the other hand, tumor-suppressive miRNAs can prevent cancer by inhibiting oncogenes and promoting processes such as apoptosis and cell cycle arrest. One of the best-studied tumor-suppressive miRNAs is miR-34a, which is often downregulated in cancers such as breast, liver, and lung cancer (Li et al., n.d.). miR-34a acts as a transcriptional target of the tumor suppressor p53, a critical regulator of the cellular stress response (Tarasov et al., 2007). In cancer, the loss of miR-34a expression contributes to tumor progression by allowing the upregulation of oncogenes such as Notch1 (Notch Receptor 1), c-MYC (Cellular myelocytomatosis oncogene), and BCL2 (B cell lymphoma 2), all of which promote cell survival and proliferation (Li et al., 2021). Restoration of miR-34a has been shown to suppress tumor growth and sensitize cancer cells to chemotherapy, indicating its potential as a therapeutic agent for cancer (Li et al., 2013; Deng et al., 2021).

Furthermore, miR-143/145 cluster has been identified as a tumor-suppressive miRNA cluster that is downregulated in various cancers, including colon cancer and bladder cancer (Bauer and Hummon, 2012; Avgeris et al., 2015). The miR-143/145 cluster inhibits cancer progression by targeting key genes involved in cell cycle regulation, apoptosis, and invasion. Specifically, miR-143 targets KRAS (Kirsten rat sarcoma viral oncogene homolog), a pivotal player in cell signaling, while miR-145 directly regulates CDC42(Cell Division Cycle 42) and RhoA (Ras Homolog Family Member A), which are crucial for the cytoskeletal organization and invasive behavior in cancer cells (Liu et al., 2019; Zhang et al., 2021). These miRNAs act to suppress cancer cell proliferation and metastasis by modulating the signaling pathways that control cellular motility and survival.

Studies have highlighted the role and therapeutic potential of miRNAs at different stages of colorectal cancer (CRC). Stage-specific miRNAs play a crucial role in CRC treatment; miR-196 regulates tumor development, progression, and response to therapy, while miR-27b-3p is involved in oxaliplatin resistance and modulates chemotherapy sensitivity through the c-Myc/miR-27b-3p/ATG10 axis. Targeting different stages of the disease, miRNA-based therapies can be enhanced through nanocarrier systems, enabling more effective and specific delivery strategies and contributing to the development of novel approaches in CRC treatment (Fadaka et al., 2023).

## 3 Nanomedicine as a platform for miRNA delivery

Although miRNAs hold great promise as therapeutic agents, their clinical application is complicated by several challenges. One of the main obstacles is their stability in the bloodstream. miRNAs are susceptible to degradation by ribonucleases in the circulation, leading to rapid clearance from the body and poor bioavailability (Balzano et al., 2015; Glinge et al., 2017). Additionally, miRNAs face difficulties in penetrating the cell membrane and entering the cytoplasm of target cells. Once inside the cell, miRNAs are often degraded by intracellular Rnases as well as double-stranded miRNAs/siRNAs are degraded by Eri1-related proteins and single-stranded miRNAs are degraded by Sdn1-related proteins reducing their therapeutic potential (Houseley and Tollervey, 2009; Chen et al., 2014).

To overcome these challenges, nanotechnology has emerged as a promising strategy for delivering miRNAs to tumors. Nanoparticles can encapsulate miRNAs, protecting them from degradation and ensuring their stability in circulation. Moreover, nanoparticles can be engineered to facilitate the targeted delivery of miRNAs to specific cancer cells, thereby enhancing therapeutic efficacy while minimizing off-target effects (Zhang et al., 2013). Nanoparticles can be functionalized with targeting ligands such as antibodies or peptides that specifically bind to overexpressed receptors on cancer cells, ensuring selective accumulation in the tumor microenvironment.

### 3.1 Types of nanoparticles used for miRNA delivery

**FIGURE 2:**
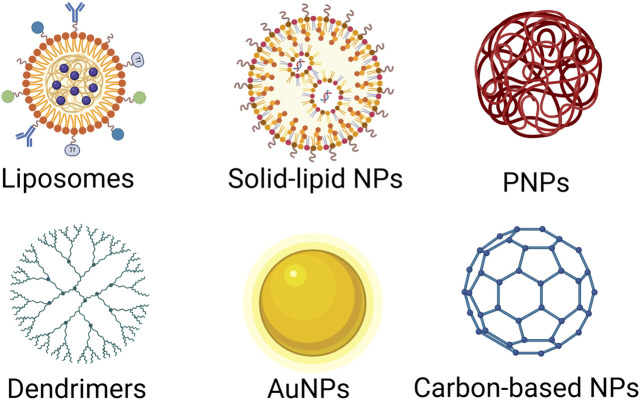
Various nanoparticle (NP) platforms used in drug delivery and nanomedicine. Lipid-Based Nanoparticles: Liposomes, composed of phospholipid bilayers and Solid Lipid Nanoparticles, composed of solid lipids, are biocompatible and commonly used for drug and gene delivery. Polymer-Based Nanoparticles: Polymeric NPs, formed from biodegradable and biocompatible polymers, are commonly used for anticancer therapy, controlled drug release, vaccines; Dendrimers, highly branched, tree-like polymers, are commonly used for gene delivery, anticancer therapy, diagnostic imaging. Inorganic Nanoparticles: Gold Nanoparticles (AuNPs), biocompatible and tunable optical properties, are commonly used for anticancer therapy, imaging drug delivery. Carbon-based nanoparticles (CNPs): have gained significant attention in nanomedicine and drug delivery due to their unique physicochemical properties, including high surface area, chemical stability, biocompatibility, and the ability to be functionalized with various biomolecules. Different NP platforms offer various advantages for drug delivery and nanomedicine, such as enhanced drug stability, targeted therapy, and controlled release. The choice of NP type depends on the drug properties, delivery route, and therapeutic goals. Created in BioRender. Telkoparan Akillilar, P. (2025) https://BioRender.com/u57v047.

Nanoparticles have gained significant attention as carriers for the delivery of therapeutic agents, including miRNAs, due to their ability to enhance stability, control release, and target specific tissues. Different types of nanoparticles offer distinct advantages based on their size, surface properties, and biocompatibility. Below are the major types of nanoparticles that have been explored for miRNA delivery in cancer therapy (Figure 2).

#### 3.1.1 Liposomes

Liposomes can be classified into different types based on their size and the complexity of their structure, such as small unilamellar vesicles (SUVs), large unilamellar vesicles (LUVs), and multilamellar vesicles (MLVs) (Lombardo and Kiselev, 2022). In the 1970s, liposomes were proposed for drug delivery, and the first liposome-encapsulated mRNA delivery systems were created in laboratories (Gregoriadis and Ryman, 1971; Hou et al., 2021; Tenchov et al., 2021). These early liposomes presented numerous challenges, including limited distribution (Sercombe et al., 2015). While some of these issues were addressed by utilizing positively charged lipids, the efficiency of these liposomes is still low and toxicity high (Silva et al., 2014; Gareev et al., 2023). Liposome formulations can be optimized for drug encapsulation efficiency, release kinetics, and stability in biological fluids. Their resemblance to biological membrane structures enhances their interaction with cells, while their biocompatibility allows for surface functionalization with ligands such as antibodies, peptides, or small molecules (Scheideler et al., 2020). The folate receptor, which is overexpressed in various cancers, including ovarian, breast, and lung cancers, is a common target for liposomal delivery systems (Dinakar et al., 2023; Gonzalez et al., 2024). Similarly, HER2-targeted liposomes have been developed for HER2-positive breast cancer therapy (Ghosh et al., 2022). Functionalizing liposomes with tumor-targeting ligands, such as folate or HER2 antibodies, have been shown to improve their ability to selectively deliver miRNAs to cancer cells (Nel et al., 2023). Liposomes can encapsulate miRNAs, protecting them from enzymatic degradation and preventing clearance by the immune system in circulation. In this case, the positively charged liposomes interact with negatively charged miRNA forming “lipoplexes” (Costa et al., 2022). However, it is important to note that a very small percentage of miRNA liposomal drug delivery systems progress to clinical trials, and the rate of failure remains high (Dasgupta and Chatterjee, 2021). Recent advances in the development of efficient and safe lipid-based delivery systems have led to the critical analysis of lipid nanoparticle (LNP) design, particularly focusing on the rationale behind their structural optimization and functionalization for targeted therapeutic applications (Mendonça et al., 2023).

#### 3.1.2 Polymeric nanoparticles

Polymeric nanoparticles (PNPs), made from biocompatible and biodegradable synthetic or natural polymers, offer several advantages in miRNA delivery. They can be engineered to provide controlled and sustained release of encapsulated miRNAs, maintaining therapeutic levels over extended periods. The biodegradability and low toxicity of PNPs make them particularly attractive for clinical applications, as they break down into non-toxic byproducts after completing their therapeutic task (Gagliardi et al., 2021).

These nanoparticles can be fabricated using materials like poly (lactic-co-glycolic acid) (PLGA), polyethylene glycol (PEG), and chitosan, all of which are known for their good biocompatibility and tunable release profiles. PNPs can also be surface-modified with tumor-targeting ligands to increase specificity towards cancer cells. For example, PLGA-based nanoparticles functionalized with folic acid have been used to target cancer cells overexpressing the folate receptor (Son et al., 2018). Moreover, PNPs can be modified with tumor-specific peptides or antibodies, such as those targeting HER2 or epidermal growth factor receptors (EGFR), further enhancing their selectivity.

One of the key innovations of polymeric nanoparticle systems is their ability to be tailored in size and surface properties, which facilitates improved tumor penetration and cellular uptake. For instance, pH-responsive PEG-functionalized nanoparticles have been used to improve the pharmacokinetics of miRNA delivery, while PLGA-based nanoparticles allow for controlled, sustained release within the tumor microenvironment (Juang et al., 2019). These nanoparticles have shown significant efficacy in preclinical models of glioblastoma and breast cancer, where they successfully delivered tumor-suppressive miRNAs like miR-34a and miR-542, resulting in reduced tumor growth and metastasis (Wang S. et al., 2016; Kapadia et al., 2020).

#### 3.1.3 Dendrimers

Dendrimers are highly branched, tree-like macromolecules that offer a well-defined, monodisperse structure. Due to their branched architecture, dendrimers provide a high surface area for the attachment of multiple miRNA molecules, targeting ligands, and other therapeutic agents. This high payload capacity makes dendrimers particularly useful for the delivery of nucleic acids, including miRNAs, in cancer therapy (Wang F. et al., 2016; Zhou et al., 2016; Zou et al., 2024). Dendrimers are typically composed of polyamidoamine (PAMAM) or polypropylene imine (PPI), both of which are biocompatible and capable of forming stable complexes with negatively charged miRNAs through electrostatic interactions.

Dendrimers offer several advantages for miRNA delivery: they are small in size, allowing for efficient cellular uptake, and their surface can be easily modified for targeted delivery. Moreover, their branching structure helps protect encapsulated miRNAs from degradation. In a study by Wang et al. (2015), dendrimer-based nanoparticles conjugated with the S6 aptamer were used to deliver miR-34a to lung cancer cells. This system, named PAM-Ap/pMiRNA-34a NPs, enhanced cellular uptake of miR-34a, which targeted autophagy-related genes such as Bcl-2 and p53 (Wang et al., 2015). The delivery system effectively inhibited tumor cell growth, migration, and invasion while promoting apoptosis, demonstrating significant therapeutic potential. The ability to fine-tune dendrimer structure also allows for the design of nanoparticles with controlled release profiles and minimal cytotoxicity, making them an attractive choice for miRNA-based therapies.

#### 3.1.4 Gold nanoparticles (AuNPs)

Gold nanoparticles (AuNPs) have attracted considerable interest as carriers for miRNA delivery due to their unique optical properties, ease of functionalization, and excellent biocompatibility (Wang et al., 2019). The surface of gold nanoparticles can be easily modified with a wide range of targeting ligands, including monoclonal antibodies, peptides, and aptamers, enabling the specific targeting of tumor cells. These modifications enhance specificity toward tumor cells and improve therapeutic outcomes. AuNPs also protect miRNAs from enzymatic degradation by RNases, thereby enhancing their stability and bioavailability in the tumor microenvironment. Additionally, gold nanoparticles have shown minimal immunogenicity, which is critical for long-term therapeutic applications. Recent studies have highlighted the therapeutic efficacy of AuNPs in cancer treatment. For example, AuNPs functionalized with PEG and loaded with miR-206 mimics have been shown to arrest breast cancer cells in the G0-G1 phase by targeting the NOTCH3 (notch receptor 3) gene (Chaudhari et al., 2022). Similarly, gold nanorods delivering miR-320a increased apoptosis and suppressed cell proliferation and metastasis in lung cancer models by enhancing PTEN (phosphatase and tensin homolog) expression and inhibiting MMP-9 (matrix metallopeptidase 9) activity (Peng et al., 2021). Additionally, the surface plasmon resonance (SPR) property of AuNPs enables real-time monitoring of miRNA binding and release, offering the potential for optimizing therapeutic protocols (Wang Q. et al., 2016). The minimal immunogenicity of AuNPs further underscores their potential for long-term applications in miRNA-based cancer therapy.

#### 3.1.5 Other nanoparticles

While liposomes, polymeric nanoparticles, dendrimers, and gold nanoparticles are the most commonly studied carriers for miRNA delivery, other types of nanoparticles, such as silica nanoparticles, carbon-based nanoparticles, and solid lipid nanoparticles, are also being explored for their potential in cancer therapy (Shi et al., 2012; Liu et al., 2016; Habib and Singh, 2022; Garrido-Cano et al., 2023). These nanoparticles offer distinct advantages in terms of stability, functionalization, and ease of synthesis.

Spray-dried LNPs exhibit optimal physicochemical and aerodynamic properties for efficient pulmonary delivery, enabling deep lung deposition and mucus penetration. These formulations have demonstrated effective gene silencing *in vitro* with high cellular compatibility and successfully silenced the housekeeping gene GAPDH in *ex vivo* human lung tissues, highlighting their potential for treating respiratory diseases such as lung cancer, asthma, COPD, cystic fibrosis, and viral infections (Zimmermann et al., 2022).

Silica nanoparticles, for instance, have high stability and can be modified with various functional groups for targeted delivery (Garrido-Cano et al., 2023). Carbon-based nanoparticles, such as graphene oxide and carbon nanotubes, exhibit unique electrical and mechanical properties that may facilitate miRNA delivery and therapeutic applications (Zhang et al., 2010).

### 3.2 Techniques for enhancing miRNA release in tumor cells

The successful delivery of miRNAs to tumor cells requires not only efficient encapsulation but also the controlled and targeted release of the miRNAs within the tumor microenvironment. Several innovative techniques have been developed to enhance the release of miRNAs from nanoparticles within tumor cells, ensuring that the therapeutic agents reach their target sites while minimizing systemic side effects (Table 1). These strategies exploit the unique characteristics of tumor environments to achieve precise, site-specific release of miRNAs.

**TABLE 1:** Techniques for Enhancing miRNA Release in Tumor Cell.

Technique	Mechanism	Triggering Stimulus	Key Advantage	Example
pH-Sensitive Nanoparticles	Nanoparticles composed of weakly acidic polymers or lipids that undergo protonation in the acidic tumor microenvironment, leading to miRNA release.	Low pH (∼6.0–6.5) typical of the tumor microenvironment (TME).	Site-specific release in acidic conditions of the tumor, reducing toxicity to normal tissues.	PEG-sedding nanoparticles encapsulating irinotecan and miR-200 for colorectal cancer treatment (Juang et al., 2019)
Enzyme-Responsive Nanoparticles	Nanoparticles engineered with enzyme-cleavable linkers that are sensitive to overexpressed enzymes like MMPs in the tumor microenvironment.	Overexpression of matrix metalloproteinases (MMPs) and other cancer-associated enzymes.	Enzyme-triggered release of miRNAs at the tumor site, minimizing leakage to healthy tissues.	MMP-sensitive nanoparticles designed to release miRNAs in response to elevated MMPs (Shahriari et al., 2019; Gonzalez-Avila et al., 2022; Egorova et al., 2023)
Thermal-Sensitive Nanoparticles	Nanoparticles that respond to localized heat, causing changes in their structure and releasing miRNA when exposed to temperature increase.	Localized heat (e.g., hyperthermia, magnetic fields, laser-induced heating).	Controlled release using external heat sources, can be combined with MRI for real-time monitoring.	Magnetic nanoparticles for tumor hyperthermia and controlled miRNA release (Yin et al., 2014; Assali et al., 2018; Peng et al., 2021)
Light-Responsive Nanoparticles	Nanoparticles designed to change structure or release profile upon exposure to specific wavelengths of light.	Light (e.g., ultraviolet or near-infrared (NIR) light).	Spatial control over miRNA delivery, NIR light can penetrate deep into tissues for activation in difficult-to-reach tumors.	Light-responsive nanoparticles using azo-dye derivatives or photo-crosslinkable polymers for controlled miRNA release (Assali et al., 2018; Liu et al., 2020, Liu et al., 2024).

#### 3.2.1 pH-sensitive nanoparticles

The tumor microenvironment (TME) is typically more acidic compared to normal tissues due to the rapid proliferation of tumor cells and the associated anaerobic metabolism (i.e., the Warburg effect). This lower pH in tumors, often around pH 6.0–6.5, contrasts with the normal extracellular pH of about 7.4. Exploiting this pH difference, pH-sensitive nanoparticles can be designed to release their miRNA cargo specifically in the acidic conditions present in tumors (Boloix et al., 2022).

These nanoparticles are usually composed of weakly acidic polymers or lipids that undergo protonation in the acidic environment of the tumor, leading to a conformational change and triggering the release of encapsulated miRNAs. For example, pH-responsive PEG-sedding nanoparticles encapsulating irinotecan and miR-200 can be engineered to release their therapeutic payload in the acidic tumor microenvironment, providing an effective approach for colorectal cancer treatment by targeting tumor growth and minimizing systemic toxicity (Juang et al., 2019).The pH-responsive delivery system ensures that miRNAs are released only in the tumor microenvironment, thereby improving selectivity and reducing off-target effects and toxicity to normal tissues.

#### 3.2.2 Enzyme-responsive nanoparticles

Certain enzymes are overexpressed in tumor tissues and play a crucial role in the remodeling of the extracellular matrix (ECM), promoting tumor invasion and metastasis. Enzyme-responsive nanoparticles are designed to exploit the presence of these enzymes to release their therapeutic payloads in tumor cells (Shahriari et al., 2019). Matrix metalloproteinases (MMPs) are one such group of enzymes that are frequently upregulated in cancer and are involved in the breakdown of the ECM, facilitating cancer cell migration and invasion.

Nanoparticles can be engineered to include enzyme-cleavable linkers that are sensitive to MMPs. When these nanoparticles encounter elevated levels of MMPs in the tumor microenvironment, the enzymes cleave the linker, triggering the release of miRNAs. This strategy allows for highly localized and enzyme-triggered release of miRNAs, ensuring that the therapeutic payload is delivered directly to cancer cells with minimal leakage to healthy tissues (Gonzalez-Avila et al., 2022).

Additionally, nanoparticles can be tailored to respond to other cancer-associated enzymes, such as cathepsins, enhancing the specificity and control over miRNA release in tumor cells (Egorova et al., 2023).

#### 3.2.3 Thermal-sensitive nanoparticles

Hyperthermia, which involves the application of localized heat to tumors, has been explored as a means to enhance drug delivery. Thermal-sensitive nanoparticles can be engineered to respond to temperature changes, releasing their miRNA cargo when exposed to heat (Assali et al., 2018). This can be achieved by applying magnetic fields or laser-induced hyperthermia to the tumor site, leading to an increase in local temperature and triggering the release of encapsulated miRNAs.

Magnetic nanoparticles, in particular, can be manipulated using an external magnetic field to generate localized heating at the tumor site. The temperature increase causes a change in the nanoparticles’ structure or stability, resulting in the release of the miRNA cargo. This technique is particularly useful in combination with magnetic resonance imaging (MRI) for real-time monitoring of drug delivery and temperature changes within the tumor (Yin et al., 2014).

Thermal-sensitive nanoparticles can also be used in combination with photothermal therapy (PTT), where nanoparticles absorb light and convert it into heat to locally increase the temperature and induce the controlled release of miRNAs (Peng et al., 2021).

#### 3.2.4 Light-responsive nanoparticles

In addition to thermal sensitivity, light-responsive nanoparticles are another exciting approach to trigger the release of miRNAs in a controlled manner. These nanoparticles can be designed to respond to specific wavelengths of light (e.g., ultraviolet or near-infrared light) to induce changes in their structure or release profile (Liu et al., 2020; Liu et al., 2024). Light-responsive materials such as azo-dye derivatives and photo-crosslinkable polymers can be incorporated into nanoparticles, allowing for precise activation of miRNA release upon light exposure. This strategy offers the advantage of spatial control over miRNA delivery, as light can be directed at specific areas of the tumor, minimizing the risk of systemic side effects. The use of near-infrared (NIR) light is particularly appealing, as it can penetrate deep into tissues, allowing for the remote activation of nanoparticles in tumors located in hard-to-reach areas (Assali et al., 2018).

## 4 Therapeutic approaches and clinical applications

The integration of miRNAs with nanomedicine has revolutionized cancer treatment strategies by enabling the precise modulation of miRNA expression. These therapies aim to either restore the function of tumor-suppressive miRNAs, inhibit the activity of oncogenic miRNAs, or modulate miRNA expression to enhance the effectiveness of conventional cancer therapies, such as chemotherapy, radiotherapy, and immunotherapy.

### 4.1 Examples of miRNA-Nanoparticle therapies in preclinical and clinical trials

Several miRNA-nanoparticle formulations have entered preclinical and clinical trials, showcasing the potential of this approach in cancer therapy (Table 2). Notable examples include:

**TABLE 2:** Therapeutic Approaches and Clinical Applications of miRNAs.

miRNA	Targeted Cancer Types	Therapeutic Effect	Clinical Trials / Preclinical Studies	References
miR-34a	Lung, breast, liver, pancreatic cancers, triple-negative breast cancer	Inhibits tumor growth, induces apoptosis, enhances chemotherapy and radiotherapy sensitivity, particularly in hepatocellular carcinoma.	Liposomal formulations of miR-34a have shown reduced tumor size and improved patient outcomes in HCC. Preclinical studies in TNBC.	Hong et al. (2020), Kapadia et al. (2020), Kardani et al. (2020), Mockly et al. (2022)
miR-21 Inhibition	Breast, liver, lung, glioblastoma, colon cancer	Reduces cancer cell proliferation, migration, and invasion, inhibits tumor growth and metastasis, especially in glioblastoma and colon cancer.	Nanoparticles loaded with anti-miR-21 reduce miR-21 expression, inhibit tumor growth and metastasis. Synergy with chemotherapy for improved efficacy.	Yan et al., (2008), Li et al. (2014); Seo et al. (2019), Zhang et al. (2020), Sun et al., (2021), Abrishami et al. (2024)
miR-155	Lymphoma, colorectal carcinoma, pancreatic cancers, breast cancer	Suppresses tumor progression, enhances sensitivity to chemotherapy (e.g., doxorubicin), particularly in breast cancer.	Inhibition of miR-155 suppresses tumor growth and enhances chemotherapy sensitivity, improving therapeutic outcomes in breast cancer.	Gironella et al. (2007), Zhang et al. (2011), Qu et al. (2015), Kardani et al. (2020), Zheng et al. (2020)
miR-221	Hepatocellular carcinoma (HCC)	Promotes tumor growth by targeting p57 (CDKN1C), p27 (CDKN1B), and BCL-2-modifying factor (BMF). Blocking miR-221 reduces tumor growth and increases survival.	A miR-221 anti-miR therapy is under development by Regulus Therapeutics and Sanofi and is in the preclinical stage.	Fornari et al. (2008), Li and Rana (2014)
Let-7	Lung cancer, breast cancer	Inhibits the RAS oncogene and reduces HMGA2 expression, suppressing tumor growth. Confirmed tumor-suppressive role in KRAS-mutant cells and lung cancer models.	Mirna Therapeutics is developing let-7 as a miRNA replacement therapy.	Reinhart et al., (2000), Johnson et al. (2005), Li and Rana (2014)

#### 4.1.1 miR-34a

miR-34a is a well-characterized tumor-suppressive miRNA that has been targeted for delivery to various cancer types, including lung, breast, liver, and pancreatic cancers. Preclinical studies have shown that miR-34a-loaded nanoparticles can inhibit tumor growth, induce apoptosis, and enhance the sensitivity of cancer cells to chemotherapy and radiotherapy. For instance, liposomal formulations of miR-34a have been shown to inhibit the progression of triple-negative breast cancer by targeting genes involved in cell cycle regulation and apoptosis (Kapadia et al., 2020). In clinical trials, miR-34a-loaded nanoparticles have demonstrated promising results, including reduced tumor size and improved overall patient outcomes, particularly in hepatocellular carcinoma (HCC) (Hong et al., 2020). However, it should be pointed out for this particular miRNA that a recent study showed that perhaps miR-34a does not have a general tumor suppressing function and that administration of synthetic miR-34a behaves similarly to existing anticancer drugs, producing cytotoxic effects. This would also allow explaining negative side effects when miR-34a mimic is administered to patients (Mockly et al., 2022; Mockly and Seitz, 2023).

#### 4.1.2 miR-21 inhibition

miR-21 is a well-established oncomiR, commonly overexpressed in a wide range of cancers, including breast, liver, lung, and glioblastoma (Yan et al., 2008; Gaur et al., 2011; Zhang et al., 2020). The inhibition of miR-21 has been shown to reduce cancer cell proliferation, migration, and invasion (Li et al., 2014). Nanoparticles loaded with anti-miR-21 have been developed to specifically target tumor cells and reduce miR-21 expression, leading to the downregulation of several oncogenic pathways. These therapies have demonstrated the potential in inhibiting tumor growth and metastasis in preclinical models, including those of glioblastoma and colon cancer (Seo et al., 2019; Abrishami et al., 2024). The combination of anti-miR-21-loaded nanoparticles with chemotherapeutic agents has also shown synergy, increased therapeutic efficacy and overcoming drug resistance (Sun et al., 2021).

#### 4.1.3 miR-155

miR-155 is another oncogenic miRNA that is upregulated in various cancers, including lymphoma, colorectal carcinoma, and pancreatic cancers (Gironella et al., 2007; Qu et al., 2015; Zheng et al., 2020). Nanoparticles delivering miR-155 inhibitors have shown significant potential in attenuating tumor growth and enhancing the efficacy of chemotherapeutic agents. Specifically, silencing miR-155 has been demonstrated to increase the sensitivity of breast cancer cells to doxorubicin by targeting critical pathways involved in DNA repair and chemotherapy resistance (Kardani et al., 2020). Preclinical investigations suggest that miR-155 inhibition not only suppresses tumor progression but also synergizes with conventional chemotherapy, leading to improved therapeutic outcomes.

#### 4.1.4 miR-221

miR-221 has been found to be frequently upregulated in human hepatocellular carcinoma (HCC) (Fornari et al., 2008). miR-221 can promote tumor growth in HCC cells by targeting the expression of p57 (CDKN1C), p27 (CDKN1B), and BCL-2-modifying factor (BMF). Blocking miR-221 has been shown to reduce tumor growth and increase survival in animal models. A miR-221 anti-miR therapy is currently being developed by Regulus Therapeutics and Sanofi and is at the preclinical stage (Li and Rana, 2014).

#### 4.1.5 Let-7

Let-7 miRNA is one of the earliest discovered miRNAs (Reinhart et al., 2000) and is associated with lung cancer by inhibiting the RAS oncogene (Johnson et al., 2005). Reduced let-7 levels lead to increased HMGA2 expression, promoting tumor growth. Its tumor-suppressive role has been confirmed in KRAS-mutant cells and lung cancer models. Let-7 also inhibits the growth of other cancers, including breast cancer. Mirna Therapeutics is developing let-7 as a miRNA replacement therapy (Li and Rana, 2014).

### 4.2 Synergistic approaches: combining miRNA delivery with other therapies

A promising strategy to enhance the therapeutic efficacy of miRNA-based cancer therapies is to combine miRNA delivery with other treatment modalities. These combinations can have synergistic effects, improving patient outcomes, overcoming resistance mechanisms, and reducing treatment-related side effects. Some key combinations include:

#### 4.2.1 Chemotherapy

miRNA-nanoparticle combinations can sensitize cancer cells to chemotherapy by regulating genes involved in drug resistance. For instance, miR-34a-loaded nanoparticles have been shown to target genes such as p53, BCL-2, and MDM2, which play key roles in chemotherapy resistance. In preclinical models, the delivery of miR-34a with doxorubicin enhanced the cytotoxic effects of the chemotherapy drug and significantly improved tumor regression (Cosco et al., 2015; Iqbal et al., 2023). This synergistic approach can also reduce the required doses of chemotherapy, thus minimizing the toxic side effects typically associated with these treatments.

#### 4.2.2 Radiotherapy

miRNAs can modulate the radio-sensitivity of cancer cells by regulating pathways involved in DNA damage repair and apoptosis. For example, miR-29 has been shown to sensitize cancer cells to radiation-induced apoptosis by inhibiting DNA repair mechanisms and promoting cell cycle arrest. The combination of miR-29-loaded nanoparticles with radiotherapy has been demonstrated to improve tumor response to radiation and reduce recurrence rates in preclinical cervical cancer (Zhang et al., 2019).

#### 4.2.3 Immunotherapy

miRNA-based therapies can also be combined with immunotherapy to boost the immune response against cancer. miRNAs such as miR-34a, miR-155, and miR-16 play important roles in regulating immune cell function. For example, miR-34a has been shown to enhance T cell activation and dendritic cell function, which can improve the effectiveness of immune checkpoint inhibitors like PD-1 and CTLA-4 inhibitors (Yadav et al., 2024).In preclinical studies, the combination of miRNA-based therapies with immune checkpoint inhibitors has resulted in improved tumor clearance and better overall survival (Cortez et al., 2019). This approach holds great promise for enhancing the efficacy of immune checkpoint inhibitors and overcoming immune evasion mechanisms in cancer.

### 4.3 Challenges

While the integration of miRNA-based therapies with nanomedicine holds significant promise for cancer treatment, several challenges must be addressed for successful clinical translation. These challenges primarily revolve around issues related to nanoparticle properties, biocompatibility, targeting efficiency, and the complexities of tumor biology.

#### 4.3.1 Current limitations

##### 4.3.1.1 Immunogenicity

One of the primary challenges associated with nanoparticle-based therapies is immunogenicity. Nanoparticles, particularly those made from synthetic materials such as lipids, polymers, or even inorganic substances, have the potential to trigger immune responses. This immunogenicity can lead to opsonization, a process where serum proteins such as immunoglobulins and complement proteins adsorb onto the nanoparticle surface, marking them for recognition and clearance by phagocytic cells such as macrophages and monocytes (Owens and Peppas, 2006).The rapid clearance of nanoparticles from the bloodstream by the mononuclear phagocyte system (MPS) reduces their circulation time, thereby limiting their accumulation in tumor tissues. This rapid elimination significantly impacts therapeutic efficacy, as the nanoparticles fail to achieve sufficient drug concentrations at the target site (Moghimi et al., 2001).

To mitigate immunogenicity and improve the biocompatibility of nanoparticles, several strategies have been developed. One widely used approach is PEGylation, where nanoparticles are coated with biocompatible polyethylene glycol (PEG). This coating forms a hydrophilic shield around the nanoparticle, reducing protein adsorption, minimizing immune system recognition, and prolonging systemic circulation time, which is crucial for therapeutic applications (Suk et al., 2015). Another innovative method involves biomimetic nanoparticles, where nanoparticles are coated with cell membrane-derived materials, such as red blood cell membranes. This approach helps the nanoparticles evade immune recognition, significantly reducing immunogenicity (Hu et al., 2011). Additionally, immune modulation strategies can be employed by incorporating immunomodulatory agents into nanoparticle formulations. These agents can suppress immune activation by downregulating macrophage activity or inhibiting complement system activation, which enhances the stability of nanoparticles in the bloodstream (Mitchell et al., 2020). Finally, size and shape optimization are crucial factors in immune evasion. Nanoparticles with a hydrodynamic diameter between 10 and 100 nm are less likely to be cleared by the mononuclear phagocyte system (MPS), allowing for more efficient circulation and reduced immune system recognition compared to larger particles (Barua and Mitragotri, 2014). These strategies collectively enhance the safety and therapeutic potential of nanoparticles in drug delivery and other biomedical applications.

##### 4.3.1.2 Toxicity

Despite significant advancements in the design and application of biocompatible nanoparticles, concerns regarding their long-term toxicity persist. Repeated administration of nanoparticles poses a risk of bioaccumulation, especially in organs like the liver, spleen, lungs, and kidneys, which are primary sites for nanoparticle uptake and clearance. This accumulation can disrupt normal organ function over time, leading to chronic toxicity. For example, studies have demonstrated that certain nanoparticles, such as those made from heavy metals (e.g., quantum dots or gold nanoparticles), can elicit oxidative stress, inflammatory responses, and even cellular apoptosis (Albalawi et al., 2021). Nanoparticles with poor biodegradability can remain trapped in these organs, potentially leading to fibrosis, inflammation, or hepatotoxicity. Additionally, nanoparticles designed for enhanced retention may inadvertently prolong exposure to toxic components, exacerbating their adverse effects.

To overcome the adverse effects associated with nanomaterials, several strategies are being explored to improve their safety profile while maintaining therapeutic efficacy. One key approach is the use of biodegradable nanoparticles, such as poly (lactic-co-glycolic acid) (PLGA) and chitosan, which degrade into non-toxic byproducts that are metabolized and excreted naturally, reducing the risk of long-term accumulation in the body (Karlsson et al., 2018). Another strategy involves surface modifications, such as PEGylation, which not only reduce immunogenicity but also minimize interactions with off-target tissues, thereby lowering the likelihood of unintended toxicity (Suk et al., 2015). Additionally, incorporating targeting ligands on the surface of nanoparticles can enhance specificity to tumor tissues, further reducing systemic exposure (Zhong et al., 2014). Size and shape optimization also play a crucial role, as smaller nanoparticles (<10 nm) are more likely to be cleared via renal pathways, minimizing accumulation in major organs. Moreover, rod-shaped or disk-shaped nanoparticles may demonstrate distinct biodistribution patterns, potentially reducing organ-specific toxicity compared to spherical nanoparticles. Lastly, the development of hybrid nanoparticles that combine biodegradable polymers with inorganic materials aims to retain therapeutic efficacy while minimizing toxicity. These hybrid platforms are often engineered to degrade into smaller, less harmful components, allowing for controlled drug release and reduced adverse effects (Parveen et al., 2023).

##### 4.3.1.3 Off-target effects

Despite significant advancements in nanoparticle-based drug delivery systems, off-target effects remain a major challenge. NPs are designed to enhance therapeutic efficacy by improving drug solubility, stability, and controlled release. However, the non-specific binding of nanoparticles to healthy cells and tissues can lead to unintended side effects, which may reduce or negate the overall therapeutic benefits. Even with the use of tumor-targeting ligands, achieving precise targeting remains difficult.

To overcome this challenge, the development of more sophisticated targeting strategies that account for the complex tumor microenvironment is necessary. Improvements in ligand selection, receptor-mediated targeting, and nanoparticle surface modifications are key areas of ongoing research (Yan et al., 2018). Additionally, designing nanoparticles that respond to specific stimuli within the tumor microenvironment, such as pH or enzymatic activity, allows for controlled drug release at the target site. For instance, pH-sensitive nanoparticles can release their payload specifically in the acidic environment typically found in tumors (Yang and Gao, 2017).

## 5 Conclusion and perspectives

The integration of microRNA-based therapies with nanomedicine represents a groundbreaking approach in the field of cancer treatment. Nanoparticles, serving as efficient delivery systems, offer a means to enhance the stability, bioavailability, and targeting of miRNAs, making it possible to restore tumor-suppressive miRNAs or inhibit oncogenic miRNAs with high precision. This innovative combination holds the potential to overcome the limitations of traditional cancer therapies and offer more precise, personalized, and effective treatments for cancer patients.

While significant challenges remain, particularly in the areas of immunogenicity, toxicity, and off-target effects, recent advances in nanoparticle design and targeting strategies have paved the way for the clinical application of miRNA-based nanomedicine. These challenges are being addressed through the development of smart nanoparticles, biodegradable materials, and personalized treatment strategies, which hold great promise for improving the therapeutic index of miRNA-based therapies.

Additionally, the synergistic combination of miRNA therapies with other treatment modalities such as chemotherapy, radiotherapy, and immunotherapy has shown substantial promise in preclinical and early clinical trials. This approach could revolutionize cancer treatment by enhancing the therapeutic effects while minimizing side effects, thus offering a comprehensive and integrated solution to the complex nature of cancer.

Looking ahead, continued research and development in the areas of nanoparticle formulations, miRNA delivery optimization, and overcoming safety and efficacy challenges will be crucial to translating miRNA-nanomedicine into a routine clinical practice. With ongoing advancements, the integration of miRNA-based therapies with nanomedicine has the potential to become a cornerstone in personalized cancer treatment, offering new hope to patients and significantly improving treatment outcomes worldwide.
